# Person-centered dementia care during COVID-19: a qualitative case study of impact on and collaborations between caregivers

**DOI:** 10.1186/s12877-022-02794-1

**Published:** 2022-02-07

**Authors:** Kana Kazawa, Ayuto Kodama, Kaoru Sugawara, Mikio Hayashi, Hidetaka Ota, Daisuke Son, Shinya Ishii

**Affiliations:** 1 Department of Medicine for Integrated Approach to Social Inclusion, Graduate School of Biomedical and Health Sciences, Hiroshima University, 1-2-3 Kasumi, Minami-ku, Hiroshima, 734-8553 Japan; 2 Advanced Research Center for Geriatric and Gerontology, Akita University, 1-1 Tegatagakuen-machi, Akita, 010-8502 Japan; 3 Center for Medical Education, Kansai Medical University, 2-5-1 Shinmachi, Hirakata, Osaka, 573-1010 Japan; 4 Department of Community-based Family Medicine, Faculty of Medicine, Tottori University, 86 Nishi machi, Yonago, Tottori, 683-8503 Japan

**Keywords:** Older people with dementia, Caregivers, COVID-19, Infection control, Person-centered care

## Abstract

**Background:**

Little is known about the actual impact of COVID-19 on caregivers of older people with dementia and resultant collaborations among them to provide continued person-centered care while undertaking infection control measures. In this study, we explored the impact of providing dementia care during COVID-19 on caregivers involved in dementia care.

**Methods:**

This is an exploratory qualitative case study. The participants were family members living with older people with dementia, care managers, and the medical and long-term care facility staff. Data were collected from 46 caregivers via face-to-face and semi-structured interviews and analyzed using thematic analysis.

**Results:**

The interviews identified 22 themes related to the impact of COVID-19 on different positions of the caregivers involved in dementia care and their collaboration, and we categorized them into six categories. The core themes were “re-acknowledgement of care priorities” and “rebuilding of relationships.” When caregivers’ perceptions were aligned in the decision-making processes regarding care priorities, “reaffirmation of trust” and “strengthening of intimate relationships” emerged as positive changes in their relationships. Furthermore, the differences in the ability of each caregiver to access and select correct and appropriate information about COVID-19, and the extent of infection spread in the region were related to “anxiety during COVID-19 pandemic” and caused a “gap in perception” regarding infection control.

**Conclusions:**

The present study clarified that the process of aligning the perceptions of caregivers to the objectives and priorities of care for older people with dementia during COVID-19 pandemic strengthened the relationships among caregivers. The findings of this study are useful for caregivers involved in person-centered dementia care.

## Background

To combat the pandemic of 2019 novel coronavirus disease (COVID-19), stringent public health measures are being implemented around the world and citizens are being forced to modify their lifestyles to accommodate measures for infection prevention [1]. Older people with dementia (PWD) are at a higher risk of infection and severity of the disease [2, 3]. COVID-19 alters their lifestyles and environment by limiting the social support they receive face-to-face, and this has an adverse impact on their cognitive and physical functions [4, 5]. Furthermore, worsening of behavioral and psychological symptoms such as apathy, anxiety, and agitation has been reported in older PWD during COVID-19 due to cognitive decline and stress pertaining to environmental changes [6, 7]. The COVID-19 pandemic affected not only the older PWD, but also their family members who provided daily care by increasing their anxiety about the risk of infection as well as the burden of care [8–10].

In Japan, COVID-19 pandemic has affected older PWD and their families [11, 12]. According to a national survey, as many as 55.0% of those who use public long-term care insurance (LTCI) services need care on a daily basis due to dementia [13]. In Japan, LTCI services were introduced to support older people with disabilities in the year 2000. It provided support or care to persons with certified disabilities depending on their physical or cognitive impairment [14]. The LTCI services include home-visit services (care, nursing, rehabilitation, etc.), day services (outpatient care, rehabilitation), short-stay services, and services at long-term care facilities. However, the use of outpatient services significantly decreased across the nation with the outbreak of the COVID-19 pandemic as preventive measures were adopted to limit gatherings [15]. Simultaneously, long-term care facilities also decided to lock down and shifted to care that followed preventive measures including social distancing and other infection control measures [16]. Therefore, the reduction or cessation of services due to the pandemic led to worsening of dementia symptoms among older PWD, and an increase in the care burden and physical and mental health problems in their families [17].

Person-centered care, which is the foundation of dementia care, respects and responds to the perspectives of the PWD, building mutually trusting relationships, and providing support to enable him/her to demonstrate their own personality and abilities [18, 19]. Caregivers have emphasized person-centered care, staying close to older PWD to carefully understand their facial expressions, conversations, behaviors, and surrounding environment. However, in the wake of COVID-19, social and physical contact between older PWD and their caregivers decreased, and it was difficult for caregivers to implement the same contact-based care as before [20]. In order to provide continued dementia care and support while adhering to infection control measures for older PWD, family members and formal caregivers such as healthcare and long-term care professionals need to seek best practices and collaborate with each other. Understanding the impact of COVID-19 pandemic on caregivers involved in dementia care, and collaborative experiences in implementation of person-centered care whenever possible, will contribute towards finding solutions to these challenges.

Regarding dementia caregivers’ experiences, qualitative works in the UK revealed difficulties in decision-making for family members for the use of in-home care services [21] and informal social support during the pandemic [22]. Initial qualitative research in India explored difficulties in caring due to reduced formal and informal social support due to preventive measures established to curb further spread of the virus, and research in Japan showed that some older PWD felt anxious about decreasing social interactions or formal care services [12]. These findings raise implications for providing better personal protective equipment for formal caregivers and maintaining relationships and providing psychological support for informal caregivers. Meanwhile, no studies have been found that focus on the relationship and collaboration between family members and formal caregivers.

This study attempts to understand the impact on different positions of caregivers, their relationships, and the reality of their collaborations in caring for older PWD during COVID-19. Qualitative research helps to understand a person’s thoughts and experiences within their cultural, social, and temporal contexts. Therefore, to fathom the impact on caregivers who were providing dementia care during COVID-19, we conducted interviews with caregivers including family members and healthcare and long-term care professionals.

It is expected that the findings from this study will be useful in considering how to support caregivers and construct a system to support them to achieve a balance between infection control and dementia care.

## Methods

This study adhered to the Standards for Reporting Qualitative Research [23]. Multiple realities are socially constructed by people, and a person’s experience is context-bound. In this study, in order to understand the participants’ experiences in their context, we used an exploratory qualitative case study methodology positioned within an interpretive paradigm [24]. The strength of this approach is that it gives due consideration to various perspectives of a phenomenon, allowing for an in-depth investigation using multiple sources and methods for data collection [25, 26].

### Participants

The participants in this study were 46 people involved in different positions in providing dementia care: family members, care managers, and staff at the medical and long-term facility for the elderly. In Japan’s LTCI system, care managers are responsible for planning and evaluating care plans and coordinating medical and long-term care services for people who require care. Authors requested the Alzheimer’s Association Japan, Care Manager Association, and medical and long-term care facilities to cooperate in the study and introduce the participants to the study. The participants were informed of the study’s purpose and all of them gave their written informed consent. Study protocols were approved by the ethical committee for epidemiology of Hiroshima University and the ethics committee of Akita University. All procedures were carried out in accordance with approved protocols and the Declaration of Helsinki.

We also considered that the severity of COVID-19 spread in different regions would affect the perceptions of residents and their living environment. For this reason, two regions (Hiroshima and Akita prefectures) with varying extents of COVID-19 spread as of August 2020 were chosen as the study field. At the time of the study, Hiroshima was in a serious situation where the number of new positive cases continued to increase, while in Akita, only a few positive cases were reported.

### Data collection and analysis

**Fig. 1:**
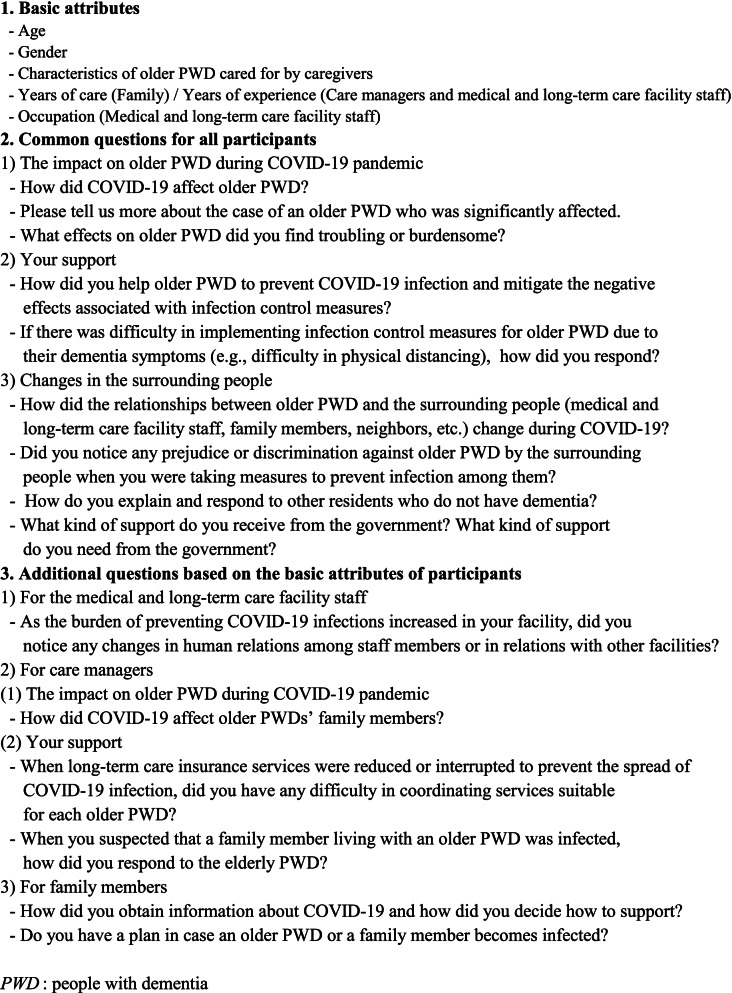
Interview guide

Three researchers who were healthcare professionals with many years of experience in dementia care conducted the interviews. Between October 2020 and February 2021, semi-structured face-to-face interviews of about 1 h each were conducted with each participant, using an interview guide prepared by the researchers (Fig. 1). The content of the interview was recorded on an IC recorder after obtaining consent from the research participant, and a verbatim transcript was prepared.

In this study, we thought that thematic analysis would be appropriate because the recurring patterns in the caregivers’ narratives helped us understand their thoughts and experiences. The interview data were thematically analyzed, including generative coding, and theorizing to identify emergent concepts in the dataset [27]. An inductive coding approach was applied by co-authors, MH and DS in several iterations until an agreement was achieved. The themes were categorized into main and subcategories and tabulated using NVivo11 (QSR International, Australia) to identify their frequency. All the authors checked the analysis. In case of disagreement, the authors discussed and reviewed the data until consensus was reached. When no new themes emerged, the researchers agreed that theoretical saturation was achieved and ended the recruitment of participants. To ensure the quality of the study, consultations were held with experts in older care, dementia care, and qualitative research to discuss the credibility and confirmability of the results.

## Results

The results were based on interviews with 46 participants, out of which four were family members, 15 were care managers, and 27 were staff at the medical and long-term facility for the elderly. The basic attributes of the participants are listed in Table 1. All family members were the primary caregivers living with the older PWD, and their years of care ranged from 1 to 18 years. The mean duration of care experience was 8.5 + 5.0 years (range 2–17 years) for care managers and 14.7 + 8.3 years (range 3–29 years) for the medical and long-term facility staff. The occupations of care facility staff were nurses, physical therapists, speech pathologists, nutritionists, care workers, consultants, and administrators.

**Table 1:** Characteristics of participants

Attributes	Family members (*n* = 4)	Care managers (*n* = 15)	Staff at the medical and long-term care facility for the elder (*n* = 27)
Region (n)			
Hiroshima	2	3	4
Akita	2	12	23
Age (n)			
20’s to 30’s	0	5	7
40’s to 60’s	2	10	20
60’s to 70’s	2	0	0
Gender (n)			
Female	3	12	17
Male	1	3	10
Characteristics of older PWD cared for by caregivers	One with mild dementia, three with severe dementia (one of whom was highly dependent on medical care)	The severity of dementia ranges from mild to severe, and the degree of medical dependence varies.	
Years of care^a^ / Years of experience^b^ [median (interquartile range)]	1, 8, 15,18, respectively	8.0 (4.5–11.5)	15.0 (7.0–20.0)
Occupation			Nurse, physical therapist, speech pathologist, nutritionist, care worker, consultant, administrator

*PWD* people with dementia

^a^Years of care (Family)

^b^Years of experience (Care managers, medical and long-term care facility staff)

From the content of the interviews, we identified 22 themes related to the response and collaboration of those involved in dementia care and categorized them into six categories (Table 2): re-acknowledgement of care priorities, rebuilding of relationships, re-recognition of trust, strengthening the close relationships, anxiety during COVID-19 pandemic, and gap in perception.

**Table 2:** Emergent themes

**Re-acknowledgement of care priorities**	
Best practice on COVID-19 restrictions	
Re-acknowledgement of individualized care continuity	
Re-confirming advanced care planning	
**Rebuilding of relationships**	
Re-acknowledgement of person-centered relationship	
Rebuilding relationships with healthcare and long-term care professionals	
Rebuilding relationships with family members	
**Re-recognition of trust**	
Trust in primary physician	
Trust in healthcare and long-term care professionals	
Trust in family members	
**Strengthening the close relationships**	
Sympathetic feelings between families	
Cohesiveness of healthcare and long-term care team	
Enhancing peer support	
**Anxiety during COVID-19 pandemic**	
Self-care to prevent infection	
Anxiety about care in long-term care facilities	
Anxiety caused by restrictions on visits to the facilities	
Anxiety about continuity of long-term care insurance services	
Conflict arising from changes in the environment of older PWD	
Caring for older PWD while taking infection control measures	
**Gap in perception**	
Gap in perception of families and healthcare and long-term care professionals	
Difficulties in agreeing upon the direction of care among healthcare and long-term care professionals	
Gap in perception of government and others	
Differences in opinion on preventing the spread of infection in the region	

*PWD* people with dementia

**Fig. 2:**
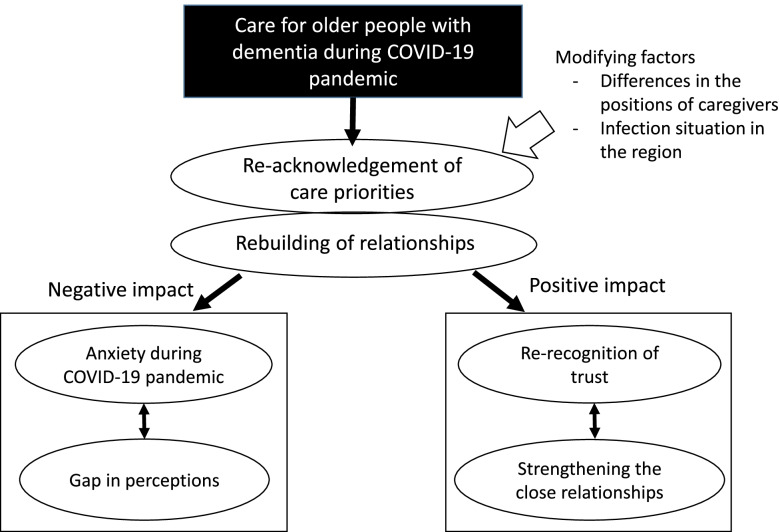
Relationships between themes

Figure 2 shows the relationships between the thematic categories. First, the participants re-acknowledged care priorities for older PWD during COVID-19, and rebuilt relationships with others involved in dementia care by re-confirming the significance of their existence and caring for older PWD. Second, relationships among caregivers were positively or negatively affected by the differences in decision-making process regarding the priority of care and the situation of infection spread in the regions.

### Re-acknowledgement of care priorities

Due to the seriousness of the COVID-19 infection, long-term care services were reduced to prevent the spread of infection among older PWD and the people around them and the outbreak of infection clusters in facilities. This forced caregivers to change their care methods which had been based on social and physical contact. With these changes in the supply and methods of long-term care services, families and formal caregivers reevaluated the purpose of care for the PWD and re-acknowledged which care should be prioritized and continued for each older PWD, focusing on their perspective. Specifically, caregivers considered how to collaborate to continue life-sustaining care such as suction and tube feeding, and daily living support. In situations where the extent of infection spread was more serious, caregivers also reconfirmed the importance of supporting the older PWD in advance care planning (ACP) of the medical and long-term care they desire while assessing their decision-making capacity. In order to ascertain the intentions of older PWD, caregivers asked them in simple terms what kind of care they would like to receive if they had serious conditions. If an old PWD was unable to express his/her own will, caregivers recognized that the best practice would be to provide care through repeated discussions between the elderly person’s family and medical professionals as his/her condition deteriorates.

Moreover, the caregivers were committed to collaborate closely, share information and assess care as appropriate.

*For a month in which the cluster occurred, the most important thing was to protect the lives of people who needed constant medical and long-term care, not just those with dementia. Advance care planning for older PWD was important. If we do not discuss it on occasion, it is difficult to decide what to choose in a critical situation (care manager, Hiroshima).*

*I am making efforts to stay in close contact with the family and other formal caregivers more than ever before (administrator of long-term care facility, Akita).*

### Rebuilding of relationships

While caring for older PWD, the caregivers were reminded of the strong relationship that had been established around older PWD. First, in this relationship, each caregiver considered his/her own roles. For example, family members felt a stronger urge to protect the lives of older PWD by being closest to them and paying close attention to infection prevention, and serving as surrogate decision-makers when necessary. Medical and long-term care facility staff sought out their roles based on their professional ethics. Second, while facing difficulties in continuing with pre-pandemic level of care, caregivers felt the significance of others who were implementing the best practices and were concerned about their physical and mental state.

*It's my life, I will take care of my wife’s life with dementia (family member, Hiroshima).*

*We frequently used short stay. However, we reduced the number of stays in order to reduce the risk of infection for PWD and users and staff of long-term care facilities. In the serious situation of infection spread, we discussed with the facility staff and took care of the person at home. I thought that the staff probably were overwhelmed with caring for older PWD while taking measures to prevent infection. (Family member, Hiroshima).*

*In a long-term care facility, family members meet a person with dementia either remotely or through a window, which may cause stress. They cannot hold hands even if they see the face (administrator of a long-term care facility, Akita).*

In addition, when the decision-making process on the priority of care was carried out and the perceptions of those involved were consistent, the positive effects included “re- recognition of trust” in others related to dementia care and “strengthening the close relationships” with those who were in the same position, such as family and peers.

### Re-recognition of trust

The attending physician and medical and long-term care facility staff shared with the families of the older PWD, detailed information about their current condition, risk of infection, and preparedness for infection, and discussed the care needed. In addition, they provided care continuously for PWD who were in locked-down long-term care facilities or were highly dependent on medical care. These commitments by the healthcare and long-term care professionals led to a re-recognition of the family members’ trust in them. Meanwhile, when the spread of COVID-19 infection became more serious, some face-to-face in-home LTCI services were reduced, and medical and long-term care staff had to monitor older PWD remotely. Therefore, family members temporarily took care of and monitored older PWD. Through this implementation of care by family members, the medical and long-term care staff felt a sense of trust in them.

*Until now, care managers had, to some extent, taken the initiative to support and protect the lives of older PWD and their family members. However, in this environment, we had no choice but to ask the family members to look after and care for older PWD (care manager, Hiroshima).*

### Strengthening the close relationships

Having reaffirmed their trust in each other, family members and medical and long-term care facility staff reiterated their appreciation for being involved in caring for older PWD while paying close attention to their own infection risks. Family members were concerned for each other’s health, empathizing with the positive and negative emotions associated with caregiving in the midst of the COVID-19 pandemic crisis. Healthcare and long-term care professionals on the frontlines were focused on communicating to encourage each other. The caregivers who continued to support the lives of older PWD also acknowledged the value of positive daily connections among them.

*My father said to me, "Try not to push yourself too hard," and his words support me (family member, Hiroshima).*

*We were stressed, so tried to maintain good relationships in the workplace. I could observe the communication among the hospital staff, encouraging each other (staff of medical care facility, Hiroshima).*

However, as the infection situation in the region got serious, the differences in the positions of caregivers increased the “anxiety during COVID-19 pandemic “ and caused a “gap in perception” regarding infection control.

### Anxiety during COVID-19 pandemic

As COVID-19 infection became more serious nationwide, caregivers practiced their own infection control measures but had various concerns owing to the large amount of media information about COVID-19. The family members were afraid to continue the use of LTCI services considering the older PWDs’ risk of infection. In one instance, one family member providing care to a person with severe dementia experienced strong anxiety about how to maintain their life. This family member was highly dependent on home visit long-term care services that were temporarily interrupted. In addition, their family members living far away could not return home to prevent each other from being infected. He also felt anxious about the impact on the older PWD due to the reduction in face-to-face interaction with them, and he felt lonely.

*First, I was confused. The day care center that we had used for approximately 14 years closed down. In particular, since there were few facilities that could care for severe levels of dementia, I was worried until I found the next facility (family member, Hiroshima).*

Medical and long-term care facility staff also experienced anxiety while gathering information on infection control and simulating how they would respond if a patient/ facility resident was diagnosed with COVID-19.

*In fact, I cannot imagine what to do when someone living in an institution is diagnosed with COVID-19. Zoning is being considered here (long-term care facility staff, Akita).*

Caregivers also struggled with incorporating social distancing and infection control into dementia care, which had previously been provided through face-to-face contact.

### Gap in perception

The caregivers felt a strong sense of “anxiety during COVID-19 pandemic” due to the differences in their positions and the extent of infection spread in the region. With the large amount of information about COVID-19, families felt a strong sense of fear and anxiety about the risk of infection due to the difficulty in accessing and selecting the correct and appropriate information. On the other hand, medical and long-term care facility staff had the ability to access and select appropriate information. Hence, medical and long-term care facility staff struggled to care for the patient without being overly fearful. Against this background, a gap in the perception of infection control emerged between family members and medical and long-term care facility staff. Medical and long-term care facility staff who provided care for older PWD on the front lines of each facility also felt a gap in perception between them and the government, which was responsible for controlling infection while managing healthcare resources for the entire region. These gaps in perception among caregivers also increased their anxiety.

As a result, they felt that there was a gap in perceptions of caregivers, the government, and the community regarding infection control measures and support for older PWD.

The families felt that there was a gap in perception between them and the medical and long-term care facility staff. Whereas the medical and long-term care facility staff felt that there was a gap in perception not only between them and the families, but also between the staff, and the whole community regarding infection control.

*Since the facility does not restrict family visiting, I am concerned that that the visits may increase the risk of infection (family member, Hiroshima).*

*It is also necessary to inform residents more about how to protect themselves appropriately, not excessively, against COVID-19 (care manager, Hiroshima).*

*If a family member is infected, we ask the government to support older PWD who cannot receive care. We have to support them somehow (care manager, Hiroshima).*

*In this area, awareness of infection control measures may not be high (administrators of long-term care facility, Akita).*

### Differences by participant characteristics and regions

As mentioned above, differences in background, such as the ability of caregivers to access and select the correct and appropriate information on COVID-19 and their occupation (role to play in infection control), led to a gap in perceptions regarding infection control among caregivers.

Furthermore, these results corroborated with the regional differences in the level of infection spread. In Hiroshima, the spread of infection was severe. Correspondingly, the critical experience of the unprecedented spread of infectious diseases led to re-confirming ACP in the “re-acknowledgement of care priorities” for older PWD. In addition, the positive and prominent theme in the form of “strengthening ties among relatives” was emerged through the joint efforts of caregivers in this crisis situation only in Hiroshima.

## Discussion

This study attempts to understand the impact of providing dementia care during COVID-19 on caregivers, including formal caregivers. With the help of semi-structured interviews with caregivers involved in caring for older PWD, the emergent core themes were “re-acknowledgement of care priorities” and “rebuilding of relationships.” The analysis of these themes revealed that the positive effects were “re-recognition of trust” and “strengthening the close relationships,” while the negative effects were “anxiety during COVID-19 pandemic” and “gap in perception.”

### Re-establishing relationships among caregivers through prioritizing care for older PWD

Many older people suffer from multiple chronic and non-curative conditions [28]. Therefore, health and long-term care professionals tend to focus on their QOL while considering priorities for usual care [29]. However, against the backdrop of the COVID-19 pandemic, care services were reduced and its providing methods were changed to prevent the spread of infection among older PWD and people around them and the occurrence of infection clusters in care facilities. In this critical situation, the caregivers focused on protecting the lives of older PWD and re-acknowledged the priorities of care. Besides, the consideration of care priorities for older PWD was influenced by their respective positions and expertise in care, in addition to the differences in knowledge of disease and care among the individuals, family members, and healthcare providers, including physicians [29, 30]. This pandemic further complicated the decisions about care priorities based on dementia symptoms and functioning, and assessment of daily lives of older PWD, which are very difficult issues, especially for family members. In particular, as the disease progresses, the difficulties in decision-making and daily living for older PWD increase, leading to conflicts and burdens for their families. In order to respect their rights and maintain the patients’ and their family caregivers’ QOL1, it is important for those involved in care to review older PWD’s will and priority of care whenever necessary. Furthermore, the results of this study reveal that when medical and long-term care staff provided individualized information that was easy for families to understand and cope with, and caregivers agreed on the priority of care, positive effects such as re-acknowledgement of care priorities and rebuilding of relationships were observed. In this scenario, family members were not only satisfied with the decisions about care, but also felt that the healthcare and long-term care professionals understood and supported older PWD and their families, which reaffirmed their trust. It is also important for families to have appropriate information about care, not only to improve their knowledge and coping skills, but also to consider how to maintain a balance between the required caregiving role and their own lives and needs [31].

Furthermore, healthcare and long-term care professionals, who were on the front lines in dealing with complex situations involving ethical issues, experienced stress and conflict [4, 32]. It could be inferred that when decision-making regarding care was based on close communication between older PWD, their families, and healthcare and long-term care professionals, it mitigated the mental burden of the staff. Furthermore, the trust of the families may have empowered them when they sometimes felt powerless in practicing dementia care while responding to an emerging infectious disease pandemic. A qualitative study on dementia care under the COVID-19 pandemic reported that family members felt stronger connections with caregivers by being involved in the care giving process [33]. In addition, the results of this study suggest that care decisions that were based on careful consideration of objectives and priorities, as well as good relationship building, had a positive impact on caregivers. This finding is particularly important to promote and strengthen relationship-building among caregivers who provide person-centered care.

### Implications for the development of support methods and systems for caregivers with / after COVID-19

The results of this study show that as the spread of infection in the community became severe, it became more difficult to access and select appropriate information (for family members), and this fueled caregivers’ anxiety about infection control.

In particular, families of older PWD who were highly dependent on medical or long-term care experienced strong anxiety. Based on the issues raised in this study, it is necessary to consider the establishment of a system that can provide continuous care to those who need medical and long-term care on a daily basis at the facility, regional, and national levels. Moreover, our results suggest that sharing the purpose of care among those involved, and carefully considering prioritization of care based on this purpose may reduce individual concerns and gaps in perception. Since this process is the foundation for developing person-centered care in critical situations, including COVID-19 pandemic, it is desirable to create guidelines and support tools to facilitate this process.

In addition, healthcare and long-term care professionals being on the front lines of care faced fear and concern about daily care practices while taking infection control measures for themselves and their care recipients [34]. There is a conflict between dementia care and infection control because preventative measures call for social distancing and wearing masks. This has resulted in transformation of communication or disruption in communicating through language and facial expressions, causing anxiety and stress in older PWD. In caring for older PWD during the COVID-19 pandemic, it is necessary to balance both these sides of care as much as possible. It is also important to provide psychological follow-up to medical and long-term care staff to reduce anxiety, conflict, and stress. For this purpose, it is important to continuously maintain a system that allows them to receive supervision from specialists in infectious diseases and dementia, and consultations with spiritual counselors as needed. Furthermore, the results of this study show that there was a gap in the perception of infection control among healthcare and long-term care professionals and the government. To reduce this gap, sharing facts and simulating countermeasures may be useful. Facilities that experienced outbursts of infections in the past should reflect on their care methods and systems and share their issues with other facilities. Facilities that have not yet experienced infection spread may need to conduct a simulation based on the shared information and be better prepared. From the perspective of making the system more robust, it would be desirable to involve not only individual facilities but also the government, which is responsible for managing care provision in the entire region.

### Limitation

The present study has a limitation. The results may not be transferable because our study was conducted in only two regions. When we designed the study, we fielded regions with different levels of infection spread. However, in regions where the level of infection spread was low at the time of the interview, fewer themes were extracted. For future research, it may be beneficial to recruit more participants by conducting interviews in regions with different levels of infection spread.

## Conclusions

We found that the process of sharing individualized information among caregivers, including family members and healthcare and long-term care professionals, and aligning their perceptions to the objectives and priorities of care for older PWD during COVID-19 pandemic reaffirmed trust and strengthened connections among caregivers. Conversely, the differences in the ability of each caregiver to access and select correct and appropriate information about COVID-19, and the extent of infection spread in the region increased the “anxiety during COVID-19 pandemic” and caused a “gap in perception” regarding infection control.

Based on these findings, it would be beneficial to promote and strengthen the relationships among caregivers who provide person-centered dementia care.

## Data Availability

The datasets generated and/or analyzed during the current study are not publicly available due the maintenance of confidentiality of our participants and declarations within the written information which participants had agreed on.

## Abbreviations

PWD: People with dementia
LTCI: Long-term care insurance
ACP: Advance care planning
